# Post-myocardial Infarction Heart Failure: A Review on Management of Drug Therapies

**DOI:** 10.7759/cureus.25745

**Published:** 2022-06-08

**Authors:** Gautam Swaroop

**Affiliations:** 1 Consultant Intervention Cardiologist, Sahara Hospital, Lucknow, IND

**Keywords:** β-blockers, soluble guanylate cyclase stimulators, sodium-glucose co-transporter-2 inhibitors, post-myocardial infarction, mineralocorticoid receptor antagonists, heart failure, angiotensin-converting enzyme inhibitors, angiotensin receptor neprilysin inhibitors

## Abstract

After myocardial infarction (MI), patients are at a greater risk of heart failure. Post-MI patients with left ventricular systolic dysfunction have a higher risk of mortality or morbidity. Strong and compelling data from randomized trials have demonstrated that drug therapies intended for preventing post-MI remodeling with neuro-hormonal inhibitors can considerably improve short- and long-term outcomes, including death, reinfarction, and worsening heart failure. This article aims at summarizing clinical data on established pharmacological therapies in treating post-MI patients with or without left ventricular systolic dysfunction (LVSD), and with or without signs and symptoms of heart failure.

## Introduction and background

Increased prevalence of cardiac risk factors ends up in increased risk of atherosclerosis and subsequently coronary artery disease (CAD). Cardiovascular diseases remain the foremost reason behind death globally and in India [1]. CAD manifests almost a decade earlier in India than in Western countries. The prevalence of heart failure (HF) is possibly on a rise in India due to the augmentation of traditional cardiovascular risk factors and the persistence of pre-transitional diseases like rheumatic cardiovascular disease, endomyocardial fibrosis, tuberculous pericardial disease, and anemias [1]. With the progression of CAD resulting in myocardial infarction (MI), there is a loss of myocardium leading to ventricular dilatation, hypertrophy, and formation of discrete collagen scar, which eventually results in cardiac remodeling and ends up in HF [2].

Several trials and studies have shown that change in remodeling parameters increases the chance of cardiovascular death and HF hospitalization with every increase in left ventricular ejection fraction (LVEF) by 10%. Improvement in cardiovascular death by 45% and HF hospitalization by 57% by every 10% decrease in ejection fraction (EF) [3].

Most of the HF therapies work on improving cardiac remodeling by reducing cardiac hypertrophy. This amounts to 1-4% in angiotensin II receptor blockers (ARBs), 4-12% in β-blockers, and 4% in mineralocorticoid receptor antagonists (MRAs). However, despite this optimal medical therapy with all the drugs, there is a chronic decline in cardiac function. The decline in cardiac function is additionally related to an increased risk of HF hospitalization and cardiovascular mortality [4].

In post-MI patients, management by drug therapy focuses on the reduction in heart rate; however, the same has not shown any improvement in the prognosis of the underlying disease. It has been seen that an increase in heart rate is additionally related to the increased progression of left ventricular (LV) remodeling in such patients; therefore, a reduction in heart rate causes improved prognosis in HF patients [5].

The acute loss of myocardium leads to an abrupt increase in cardiac loading conditions that induces a singular pattern of remodeling involving the infarcted border zone and remote non-infarcted myocardium. Myocyte necrosis and the resultant increase in load trigger a cascade of biochemical intracellular signaling processes that initiates and subsequently modulates reparative changes, which include dilatation, hypertrophy, and therefore the formation of a discrete collagen scar. European Society of Cardiology (ESC) guidelines for the management of chronic coronary syndrome and HF stresses the importance of guideline-directed medical therapy (GDMT) in treating patients with CAD and HF who are having reduced EF. Also, the studies have indicated that the use of GDMT post-re-vascular implementation is especially low after coronary artery bypass graft [6,7,8].

HF complicating acute MI is frequent. Among patients with acute MI, HF is the most powerful predictor of death and its important implications for treatment. This subset has even a powerful relationship between the degree of HF and mortality has been reported. The optimal management of the patient with HF complicating MI varies per time since the onset of infarction.

## Review

Incidence of HF among patients hospitalized for an acute MI varies among studies, starting from 14-36%. HF on admission increased the incidence of in-hospital mortality by 2.2 times among 13,707 patients enrolled within the Global Registry of Acute Coronary Events (GRACE) from 1999 to 2001 [9]. During the index MI hospitalization, 2,831 (37%) MI patients were diagnosed with new HF and 1,024 (13%) died. Among hospital survivors who failed to have HF during their index hospitalization (n = 4,291), a further 3,040 patients (71%) developed HF by five years, 64% of which occurred within the first year.

The estimated prevalence of HF is approximately 1% of the whole population or 8-10 million individuals. Projecting these figures to India gives an approximate number of 21 million Indians above the age of 19 who are likely to possess a history of MI. It is estimated that 10-40% of people with MI develop HF. This may mean that the likely HF burden thanks to MI in India would be 2.1-8.4 million with an affordable estimate of about 4-5 million [10]. MI remains the foremost common reason for HF worldwide.

Patients with LVEF of 35% were enrolled in the Studies of LV Dysfunction Treatment (SOLVD) trial [11], wherein patients with prior MI had a two-fold higher hospitalization rate for decompensated HF and a four-fold higher fatality rate as compared to those without a previous MI. These findings were confirmed by the Survival and Ventricular Enlargement (SAVE) trial, which found a 70% increase in the risk of cardiovascular-related death and LV enlargement in patients with left ventricular systolic dysfunction (LVSD) because of prior MI vs. those without prior MI [12].

During the first phase of CVD, (i.e., before the occurrence of structural changes within the heart, that is, infarction), the increased pulse rate may be a marker of CAD, suggesting progression of pathology. Reduction of pulse rate under these circumstances does not improve prognosis but can reduce the symptoms (angina) of the underlying disease. However, within the setting of reduced ventricular function, a rise in the pulse isn't only a marker, but also a risk factor and contributes to the progression of LV remodeling. In patients with HF, therefore, vital sign reduction is related to improved prognosis.

The loss of myocardial muscle post-MI causes the reworking within the impact zone and the ideally related to that. The second state of the myocardial muscle leads to the triggering of assorted intracellular and biochemical processes which causes ventricular dilation hypertrophy and the formation of scars. This process continues over a period of time increasing the preload and afterload and causing ventricular remodeling.

**Figure 1:**
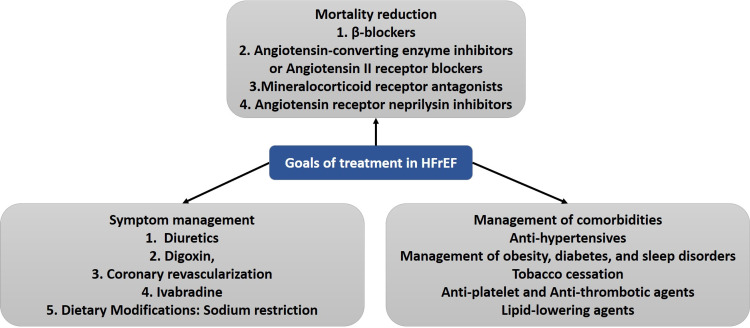
Treatment goals in patients with heart failure with reduced ejection fraction

Despite significant improvement in the drug therapies over the period of three decades, medical therapy with angiotensin-converting enzyme inhibitors (ACEIs) or ARBs, β-blockers, and MRAs. Many HF patients with reduced EF (HFrEF) significantly increased the risk for HF hospitalization and cardiovascular mortality in patients with a history of MI. Treatment goals in HFrEF patients are demonstrated in Figure 1.

Cardiac remodeling occurs in around 30% of anterior infarcts despite timely primary percutaneous coronary intervention (PCI) and the use of drugs, for example, ACEIs or ARBs, β-blockers, aldosterone inhibitors, and statins. Major trials demonstrating the mortality benefits of medical therapy in patients with HFrEF are presented in Table 1.

**Table 1:** Major trials demonstrating mortality benefit with medical therapy in patients with heart failure with reduced ejection fraction † ACEIs - angiotensin-converting enzyme inhibitors; ARBs - angiotensin II receptor blockers; ARNIs - angiotensin receptor-neprilysin inhibitors; MRAs - mineralocorticoid receptor antagonists; AIRE - acute infarction ramipril efficacy; EPHESUS - eplerenone post-acute myocardial infarction heart failure efficacy and survival study; MERIT-HF - metoprolol CR/XL randomized intervention trial in congestive heart failure; OPTIMAAL - Optimal trial in myocardial infarction with angiotensin II antagonist losartan; PARADIGM-HF - prospective comparison of ARNIs with ACEIs to determine impact on global mortality and morbidity in heart failure; SAVE - survival and ventricular enlargement; TRACE - trandolapril cardiac evaluation; and VALIANT - valsartan in acute myocardial infarction.

Medication	Class	Trial name	Enrolled patients	Primary outcome
Carvedilol	β-blockers	CAPRICORN [13]	1959	All-cause mortality or hospital admission for cardiovascular problem
Metoprolol XL	β-blockers	MERIT-HF [14]	3991	All-cause mortality
Trandolapril	ACEIs	TRACE [15]	1749	All-cause mortality
Captopril	ACEIs	SAVE [12]	2231	All-cause mortality, cardiovascular mortality, or morbidity
Ramipril	ACEIs	AIRE [16]	2006	All-cause mortality
Losartan	ARBs	OPTIMAAL [17]	5477	All-cause mortality
Valsartan	ARBs	VALIANT [18]	14, 703	All-cause mortality
Eplerenone	MRAs	EPHESUS [19]	6632	All-cause mortality, cardiovascular mortality, heart failure hospitalizations, acute myocardial infarction, stroke, or ventricular arrhythmias
Valsartan-sacubitril	ARNIs	PARADIGM-HF [20]	8442	Composite death from cardiovascular causes or hospitalizations for heart failure

Evidence with β-blockers

β-blockers have shown significant improvement in mortality and morbidity in patients with HFrEF irrespective of ischemia or non-ischemic cardiomyopathy. In most of the trials on β-blockers, patients with prior history of MI or having recent PCI have been excluded. In the CAPRICORN trial of carvedilol [13], a multicenter placebo control trial, in which 1959 patients with an acute history of MI and LVEF <40% were randomized. The beneficial effect of β-blockers in addition to those of evidence-based treatment for acute MI, including ACEIs have been seen although there was no difference between the primary endpoint; however, all-cause mortality was lower in the carvedilol group as compared to placebo. Cardiovascular mortality, nonfatal MI, and all-cause mortality or nonfatal MI were also lower in the carvedilol group as compared to the placebo. In another randomized possible control trial of carvedilol in patients of congestive HF because of ischemic heart disease in 415 patients over a period of six months and 12 months, the beneficial effect of carvedilol on left ventricle EF and sizes were maintained for at least a year after the start of treatment. However, carvedilol has no effect on exercise performance symptoms and episodes of worsening HF, and there was an overall reduction in cardiovascular events. In a different subgroup analysis of Metoprolol CR/XL Randomized Intervention Trial in Congestive Heart Failure (MERIT-HF) trial [14] of metoprolol, similar findings in such types of patients were observed. β-blockers therapy has shown a reduction in mortality in patients who are having a history of an acute coronary syndrome (ACS) or acute MI and HFrEF has been given class I recommendation in both American College of Cardiology (ACC)/ ESC guidelines for HF management. It is also been observed that there are few patients only who can reach the target dose mostly because of lower blood pressure.

Evidence with angiotensin-converting enzyme inhibitors (ACEIs)

Functional improvement and mortality benefits with ACEIs have been observed in various trials but these trials have not enrolled the patient with ischemic cardiomyopathy. In the TRAndolapril Cardiac Evaluation (TRACE) trial [15] of Trandolapril vs. placebo in post-ACS patients having LVEF <35% has shown a long-term reduction in risk of mortality and morbidity from cardiovascular causes, sudden death, and HF development. Similarly, in SAVE trial [12], there was a 22% relative risk reduction in HF hospitalization and a 25% reduction in recurrent MI with captopril vs. placebo. In the acute infarction ramipril efficacy (AIRE) trial [16], there was a 27% reduction in mortality with ramipril vs. placebo. In both these trials, post-MI patients with LV dysfunction were enrolled. American College of Cardiology (ACC) and ESC guidelines have recommended Class 1 and level A evidence to ACEIs in patients with a history of MI with LVEF.

Evidence with angiotensin II receptor blockers (ARBs)

Another renin-angiotensin-aldosterone system (RAAS) blockers i.e ARBs work at a final common pathway that is blocking angiotensin II effects. In the optimal trial of Losartan, which has randomized approximately 5000 patients with acute MI and LVEF <40%, there was no difference in all-cause mortality over 2.7 years of follow-up. The VALsartan In Acute myocardial iNfarcTion (VALIANT) trial [18] of valsartan compared outcomes with (a) ACEIs with the reference agent captopril (b) ARBs with valsartan, or (c) both in patients with HF and/or LVEF ≤40% after MI. The results showed an increase in adverse events in the combination of valsartan and captopril group as compared to captopril and valsartan alone. This trial also shows the evidence that targeting RAAS in post-MI patients with LVEF ≤40% reduces the risk of cardiovascular events. HF guidelines recommend the use of ARBs in patients with MI or ACS and having LVEF <40% who are intolerant to ACEIs in reducing the risk of mortality and morbidity (13).

Mineralocorticoid receptor antagonists (MRAs)

Evidence supports the use of MRAs in patients with New York Heart Association (NYHA) class II-IV HFrEF to reduce morbidity and mortality, including the subset of patients with ischemic cardiomyopathy. The Eplerenone Post-acute MI HF Efficacy and Survival Study (EPHESUS) [19] randomized ≈6000 patients with HFrEF to eplerenone or placebo at 16 months, eplerenone was associated with a 15% reduction in all-cause mortality and a 35% reduction in the risk of hospitalization for worsening HF may be attributable to the ability of spironolactone to reduce myocardial and vascular fibrosis. Blockade of aldosterone receptors by spironolactone, in addition to standard HF therapy, should be considered for the treatment of patients with severe HF. To gain insight into the benefit of MRAs early after MI and the development of HF symptoms, EPHESUS evaluated outcomes at a 30-day follow-up. When eplerenone was initiated about one-week post-MI in patients with HFrEF, there was a reduction in 30-day all-cause mortality. These data have been extrapolated for use of other MRAs such as spironolactone given the similar efficacy of both agents. In patients with HFrEF due to ischemic cardiomyopathy, the use of spironolactone has been associated with reversal of negative cardiac remodeling as well as decreased ventricular arrhythmias, thereby reducing morbidity risk. Reduction in mortality and morbidity with spironolactone was also seen in a large, randomized placebo-control trial, where the majority of the patients had HFrEF secondary to ischemic cardiomyopathy. According to ESC and ACC guidelines, the MRAs have been designated a Class II recommendation, with a level of evidence B in patients with LVEF ≤35% and NYHA class II-IV HF, unless otherwise contraindicated. 

Angiotensin receptor-neprilysin inhibitors (ARNIs)

Angiotensin receptor-neprilysin inhibitor (ARNIs) has the benefit of two anti-hypertensive drugs (sacubitril and valsartan). Sacubitril on one hand inhibits the degradation of natriuretic peptides (e.g., atrial natriuretic peptide (ANP), brain (or B-type) natriuretic peptide (BNP), and C-type natriuretic peptide (CNP) which have many beneficial effects. Valsartan on another hand is ARBs that inhibit the angiotensin receptor pathway. Both molecules help in reducing the chance of cardiovascular events. The paradigm HF trial [20] evaluated ARNIs vs. enalapril in HFrEF patients (LVEF ≤40%). Unfortunately, this study was prematurely stopped because of overwhelming benefits within the sacubitril arm. Further sub-analysis has suggested that there's no difference in endpoint between the ischemic and non-ischemic subgroups. The advantage of ARNIs during the post-MI period has been suggested in animal studies whereas in Prospective ARNIs vs. ACEIs Trial to see superiority in reducing HF events after MI trial carried out in post-MI patients with new LVSD dysfunction (PARADISE-MI) haven't shown any significant difference between the sacubitril/valsartan and ramipril arm.

Soluble guanylate cyclase (sGC) stimulators

In patients with HFrEF, there's a reduction in nitric oxide bioavailability that further reduces cyclic guanosine monophosphate (cGMP) synthesis. The reduction in cGMP is related to endothelial dysfunction, microvascular disease, and myocardial dysfunction. Established neuro-hormonal inhibitory therapies don't target this pathway. Vericiguat, a completely unique soluble guanylate cyclase (sGC) stimulator, appears well-tolerated and effective in reducing the N-terminal (NT)-prohormone BNP (NT-proBNP) during phase IIB multicentric clinical trial. The efficacy and safety of vericiguat are being evaluated in phase III clinical trials. Vericiguat Global Study in Subjects with HFrEF (VICTORIA) [21] trial of 4872 patients with HFrEF. The results of this trial have shown a 10% reduction in primary endpoint vs. placebo; however, a separate sub-analysis on the ischemic vs. non-ischemic group continues to be awaiting.

Sodium-glucose co-transporter-2 (SGLT2) inhibitors

In patients with symptomatic HF, SGLT2 inhibitors have shown a promising lead to reducing both composites cardiovascular death and HF hospitalization. This has been shown within the recently concluded trials of dapagliflozin, empagliflozin, and canagliflozin in patients with or without diabetes. In the Dapagliflozin and Prevention of Adverse Outcomes in Heart Failure (DAPA-HF) trial [22], the patient with etiology of ischemic cardiomyopathy were 56% and those with non-ischemic cardiomyopathy were 36%, the relative risk reduction of 36%, and the absolute difference of 4.9%, the number needed to treat (NNT) was 21. Cardiovascular death has been reduced by 18% and HF hospitalization is reduced by 30% among those that received dapagliflozin as compared to those that received placebo, regardless of the presence or absence of diabetes. Similarly in Empagliflozin Outcome Trial in Patients with Chronic Heart Failure (EMPEROR HF) trial [23], reduced patients of ischemic cardiomyopathy and non-ischemic cardiomyopathies were 52.8% and 47.2%, respectively and relative risk was reduced by 24.7% in a primary composite endpoint.

## Conclusions

Most of the HF therapies have shown a reduction in the risk of cardiovascular death and HF hospitalization in patients with post-MI ACS with LVEF <35% from 4-12%. ARNIs have also shown significant improvement in reduction in risk of cardiovascular death and HF hospitalization in patients with post-MI ACS with LVEF <35% amounting to 20% in paradigm HF trial. Newer drugs for HF like vericiguat and SGLT-2 inhibitors have shown promising results in HF trials.
